# The prognostic value of an age-adjusted BIG score in adult patients with traumatic brain injury

**DOI:** 10.3389/fneur.2023.1272994

**Published:** 2023-11-02

**Authors:** Xue Bai, Ruoran Wang, Cuomaoji Zhang, Dingke Wen, Lu Ma, Min He

**Affiliations:** ^1^Department of Critical Care Medicine, West China Hospital, Sichuan University, Chengdu, Sichuan, China; ^2^Department of Neurosurgery, West China Hospital, Sichuan University, Chengdu, Sichuan, China; ^3^Department of Anesthesiology, Affiliated Sport Hospital of Chengdu Sport University, Chengdu, Sichuan, China

**Keywords:** BIG score, traumatic brain injury, prognosis, trauma score, adult

## Abstract

**Background:**

The base deficit, international normalized ratio, and Glasgow Coma Scale (BIG) score was previously developed to predict the outcomes of pediatric trauma patients. We designed this study to explore and improve the prognostic value of the BIG score in adult patients with traumatic brain injury (TBI).

**Methods:**

Adult patients diagnosed with TBI in a public critical care database were included in this observational study. The BIG score was calculated based on the Glasgow Coma Scale (GCS), the international normalized ratio (INR), and the base deficit. Logistic regression analysis was performed to confirm the association between the BIG score and the outcome of included patients. Receiver operating characteristic (ROC) curves were drawn to evaluate the prognostic value of the BIG score and novel constructed models.

**Results:**

In total, 1,034 TBI patients were included in this study with a mortality of 22.8%. Non-survivors had higher BIG scores than survivors (*p* < 0.001). The results of multivariable logistic regression analysis showed that age (*p* < 0.001), pulse oxygen saturation (SpO_2_) (*p* = 0.032), glucose (*p* = 0.015), hemoglobin (*p* = 0.047), BIG score (*p* < 0.001), subarachnoid hemorrhage (*p* = 0.013), and intracerebral hematoma (*p* = 0.001) were associated with in-hospital mortality of included patients. The AUC (area under the ROC curves) of the BIG score was 0.669, which was not as high as in previous pediatric trauma cohorts. However, combining the BIG score with age increased the AUC to 0.764. The prognostic model composed of significant factors including BIG had the highest AUC of 0.786.

**Conclusion:**

The age-adjusted BIG score is superior to the original BIG score in predicting mortality of adult TBI patients. The prognostic model incorporating the BIG score is beneficial for clinicians, aiding them in making early triage and treatment decisions in adult TBI patients.

## Introduction

As the leading cause of mortality and disability in trauma patients, traumatic brain injury (TBI) brings damage to victims and their families' quality of life and economic burden to society. A recent study estimated that nearly 69 million individuals are diagnosed with TBI annually in the world (1). The high mortality of TBI patients makes early triage and clinical intervention extremely important. Many trauma scores have been developed to assess the injury severity of TBI and to predict the outcome of trauma patients, such as the revised trauma score (RTS), the injury severity score (ISS), and the comprehensive trauma revised injury severity score (TRISS) (2–5). Specific scoring systems aimed at predicting the outcome of TBI patients were also developed and validated, including the CRASH model and IMPACT model (6, 7). However, these scores composed of radiologic characteristics and other anatomical factors are too complex and time-consuming for clinicians making patient triage and treatment decisions in the early stage after initial brain injury.

The BIG (composed of base deficit, international normalized ratio, and Glasgow Coma Scale) score is a pediatric trauma score that was initially developed to assess children facing military and civilian traumatic injuries (8). It has been proven to accurately predict the mortality rate of pediatric trauma patients admitted to military trauma systems. Several subsequent studies externally confirmed the good performance of the BIG score in predicting the mortality of similar pediatric trauma patients (9–11). Moreover, one study confirmed that the BIG score in admission was associated with functional outcomes at hospital discharge in pediatric TBI patients (12). The BIG score performed better than other pediatric trauma scoring systems and was validated with similar accuracy in a separate pediatric population (8). A BIG score of <12 points suggests a mortality of <5%, whereas a cutoff of >26 points corresponds to a mortality of >50% (13). In addition, researchers also explored the prognostic value of the BIG score in non-specific adult trauma patients and found that the BIG score had a comparable predictive performance with TRISS and the probability of survival (PS09) score (14). Given that aging is a factor that affects the prognosis of trauma patients, we proposed to establish an age-adjusted BIG score to better predict the mortality of patients with trauma.

It was mentioned in all the above studies that the superiority of the BIG score was its availability and simplicity. Based on these findings, we designed this study to explore the prognostic value of the BIG score and compared it with other trauma triage scores in homogeneous adult TBI patients.

## Materials and methods

### Data source

This observational study was performed using data from the Multiparameter Intelligent Monitoring in Intensive Care Database III (MIMIC-III database), which was a large critical care database including patients admitted to ICUs (intensive care unit) of the Beth Israel Deaconess Medical Center between 2001 and 2012. This freely available database was approved by the Institutional Review Boards of Beth Israel Deaconess Medical Center and the Massachusetts Institute of Technology (MIT). All data of participants in this public database were deidentified and anonymized. We obtained access to utilize data from the MIMIC III database after passing the National Institutes of Health (NIH) web-based training course and the Protecting Human Research Participants examination. All needed data, including age, sex, vital signs, laboratory tests, diagnoses, length of hospital stay, records of operation, and blood transfusion of this study were extracted by us using Navicat Premium 12 (PremiumSoft, Hong Kong). The BIG score was calculated by base deficit + 2.5 × INR + (15-GCS). The computing methods of other trauma scoring systems, including RTS, new trauma score (NTS), and GCS, age, and systolic arterial pressure score (GAP), were referred to in previous studies (15, 16). The primary outcome of this study was in-hospital mortality.

### Participants

**Figure 1:**
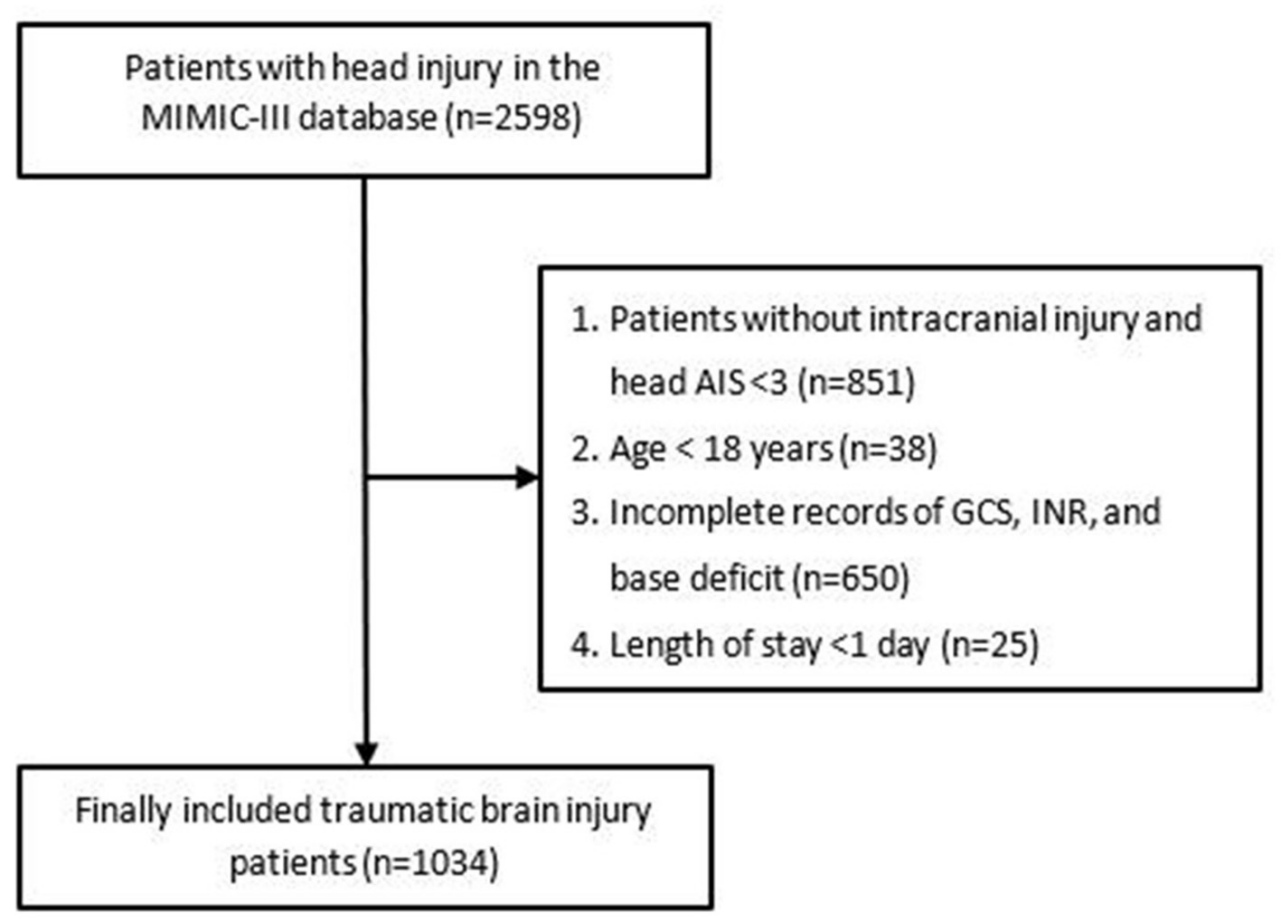
Flowchart of patients' inclusion.

Patients with head injuries from the MIMIC-III database were enrolled for this study based on ICD-9 codes (800.00–801.99; 803.00–804.99; 850.0–854.19). However, patients were excluded from this study if they met any one of the following criteria: (1) diagnosed only with extracranial injury including scalp injury and skull fracture and head AIS <3; (2) age <18 years; (3) incomplete records of GCS, INR, and base deficit; (4) discharged within 24 h following admission (due to the hasty process, these patients did not receive standard medical treatment and examination, so they lacked many related variables). After exclusion, a total of 1,034 patients were included in the final cohort. The complete flowchart of participant inclusion is shown in Figure 1.

### Statistical analysis

We utilized Kolmogorov–Smirnov tests to verify the normality of included variables. All continuous variables included in this study were expressed as median (interquartile range) and differences between groups of continuous variables were testified by the Mann–Whitney *U*-test because of their non-normal distribution. Categorical variables were expressed as numbers (percentage) and differences between groups of categorical variables were analyzed by the chi-square test. Univariable logistic regression was performed first and then, stepwise multivariable logistic regression with the entry method including significant variables in the univariable logistic regression was sequentially performed to explore the independent relationship between BIG score, other risk factors, and in-hospital mortality of included patients. The odds ratio (OR) and 95% confidence intervals (CI) of each factor were also shown. Then, multivariable logistic regression analysis was also performed to construct an age-adjusted BIG score and the multi-factor prognostic model. The nomogram of this multi-factor prognostic model was drawn for convenient clinical use. A calibration plot was drawn to evaluate the fit of the multi-factor prognostic model. Receiver operating characteristic (ROC) curves were drawn to evaluate the discriminatory ability to predict outcomes of included patients. The Youden index was used to identify cutoff values. We used the *Z*-test to compare the predictive values between factors and models.

A *P*-value <0.05 was considered to be statistically significant. We used SPSS 22.0 Windows software (SPSS, Inc, Chicago, IL) and R (version 3.6.1; R Foundation) for all statistical analyses and for drawing the figures.

## Results

### Baseline comparison of included TBI patients based on in-hospital outcomes

A total of 1,034 patients were included in this study with 798 survivors and 236 non-survivors (Table 1). The mortality rate and male ratio of included patients were 22.8 and 59.7%, respectively. The incidence of diabetes mellitus (*p* = 0.015) and hypertension (*p* = 0.004) were both higher among non-survivors. Vital signs including systolic blood pressure, diastolic blood pressure, and respiratory rate were not different between survivors and non-survivors. However, non-survivors had significantly lower heart rate (*p* = 0.032). Pulse oxygen saturation (SpO_2_) did not differ between those two groups, whereas the GCS score of non-survivors was lower than that of survivors with statistical significance (*p* < 0.001). Results of laboratory tests showed that non-survivors had a higher level of blood glucose (*p* < 0.001), base deficit (*p* = 0.047), and INR (*p* < 0.001), while the level of hemoglobin (*p* < 0.001) and platelet (*p* < 0.001) were significantly lower in non-survivors. Furthermore, the BIG score of non-survivors was significantly higher than that of survivors (*p* < 0.001), whereas survivors had a higher score of RTS (*p* < 0.001), NTS (*p* < 0.001), and GAP (*p* < 0.001). Results of injury types presented that non-survivors were more frequently diagnosed with subarachnoid hemorrhage (*p* = 0.005) and intracerebral hematoma (*p* = 0.012).

**Table 1:** Baseline comparison of TBI patients divided by the survival status.

**Variables**	**Total patients (*N* = 1,034)**	**Survivors (798, 77.2%)**	**Non-survivors (236, 22.8%)**	***p***
Age (years)	67 (46–81)	63 (42–80)	77 (62–86)	<0.001
Male gender (%)	617 (59.7%)	489 (61.3%)	128 (54.2%)	0.053
**Comorbidities**				
Diabetes mellitus (%)	82 (17.6%)	128 (16.0%)	54 (22.9%)	0.015
Hypertension (%)	399 (38.6%)	289 (36.2%)	110 (46.6%)	0.004
Hyperlipidemia (%)	126 (12.2%)	95 (11.9%)	31 (13.1%)	0.612
Cerebral vascular disease (%)	17 (1.6%)	12 (1.5%)	5 (2.1%)	0.514
Coronary heart disease (%)	148 (14.3%)	109 (13.7%)	39 (16.5%)	0.269
**Vital sings in admission**				
Systolic blood pressure (mmHg)	128 (118–147)	128 (118–146)	128 (119–149)	0.565
Diastolic blood pressure (mmHg)	64 (56–75)	64 (57–75)	64 (51–74)	0.169
Heart rate (min^−1^)	84 (72–99)	85 (73–99)	82 (71–95)	0.032
Respiratory rate (min^−1^)	18 (15–21)	18 (15–21)	18 (15–21)	0.751
SpO_2_ (%)	99 (97–100)	99 (97–100)	99 (97–100)	0.347
GCS in admission	8 (6–14)	10 (6–14)	6 (3–9)	<0.001
**Laboratory tests**				
Glucose (mg/dL)	139 (114–174)	134 (112–165)	159 (131–193)	<0.001
Hemoglobin (g/dL)	12.5 (11.1–13.8)	12.7 (11.4–14.0)	12 (10.3–13.1)	<0.001
Platelet ( × 10^9^/L)	224 (176–282)	227 (183–284)	211 (156–260)	<0.001
pH	7.38 (7.32–7.44)	7.38 (7.32–7.44)	7.38 (7.31–7.45)	0.958
Base deficit (mmol/L)	0 (−2 to 3)	0 (−2 to 3)	0 (−2 to 4)	0.047
INR	1.1 (1.1–1.3)	1.1 (1.1–1.3)	1.2 (1.1–1.7)	<0.001
BIG score	10 (5–15)	9 (4–14)	13 (9–18)	<0.001
RTS score	10 (10–11)	10 (10–12)	10 (8–10)	<0.001
NTS score	15 (13–19)	15 (14–20)	14 (11–15)	<0.001
GAP score	15 (12–19)	15 (13–19)	12 (10–15)	<0.001
Surgical intervention (%)	310 (30.0%)	231 (28.9%)	79 33.5%)	0.182
Blood transfusion (%)	457 (44.2%)	343 (43.0%)	114 (48.3%)	0.148
**Injury classification**				
Epidural hematoma (%)	20 (1.9%)	17 (2.1%)	3 (1.3%)	0.591
Subdural hematoma (%)	444 (42.9%)	333 (41.7%)	111 (32.6%)	0.149
Subarachnoid hemorrhage (%)	264 (25.5%)	187 (23.4%)	77 (32.6%)	0.005
Intracerebral hematoma (%)	144 (13.9%)	99 (12.4%)	45 (19.1%)	0.012
Length of ICU stay (days)	3 (2–7)	3 (2–7)	4 (2–7)	0.587
Length of hospital stay (days)	9 (5–15)	10 (6–17)	5 (2–10)	<0.001

SpO_2_, pulse oxygen saturation; GCS, Glasgow Coma Scale; INR, International Normalized Ratio; RTS, Revised New Trauma Score; NTS, New Trauma Score; GAP, The GCS; Age and Systolic Arterial Pressure score.

### Univariate and multivariable logistic regression analysis of risk factors for in-hospital mortality of included TBI patients

Results of univariate logistic regression analysis indicated that age (*p* < 0.001), diabetes mellitus (*p* = 0.016), hypertension (*p* = 0.004), glucose (*p* < 0.001), BIG score (*p* < 0.001), occurrence of subarachnoid hemorrhage (*p* = 0.005), and intracerebral hematoma (*p* = 0.010) were positively associated with poor outcomes of included patients (Table 2), whereas heart rate (*p* = 0.026), SpO_2_ (*p* = 0.015), hemoglobin (*p* < 0.001), and platelet (*p* = 0.046) were inversely related with poor in-hospital outcomes. All variables were then included in multivariable analysis. After adjusting confounded factors, age (*p* < 0.001), SpO_2_ (*p* = 0.032), glucose (*p* = 0.015), hemoglobin (*p* = 0.047), BIG score (*p* < 0.001), subarachnoid hemorrhage (*p* = 0.013), and intracerebral hematoma (*p* = 0.001) were still correlated with in-hospital mortality of included TBI patients.

**Table 2:** Univariate and multivariable analysis of risk factors for in-hospital mortality in included TBI patients.

**Variables**	**Unadjusted analysis**			**Adjusted analysis**		
	**OR**	**95%CI**	***p***	**OR**	**95%CI**	***p***
Age	1.029	1.021–1.038	<0.001	1.039	1.028–1.050	<0.001
Male gender	0.749	0.559–1.004	0.053			
Diabetes mellitus	1.553	1.086–2.221	0.016	0.991	0.643–1.511	0.969
Hypertension	1.538	1.146–2.062	0.004	1.186	0.832–1.692	0.344
Hyperlipidemia	1.119	0.725–1.728	0.612			
Cerebral vascular disease	1.418	0.494–4.066	0.516			
Coronary heart disease	1.251	0.840–1.864	0.270			
Systolic blood pressure	1.000	0.996–1.004	0.924			
Diastolic blood pressure	0.997	0.990–1.004	0.347			
Heart rate	0.992	0.985–0.999	0.026	0.995	0.986–1.003	0.207
Respiratory rate	0.997	0.968–1.026	0.825			
SpO_2_	0.953	0.917–0.991	0.015	0.956	0.912–0.992	0.032
Glucose	1.006	1.004–1.009	<0.001	1.003	1.001–1.006	0.015
Hemoglobin	0.836	0.781–0.894	<0.001	0.921	0.849–0.999	0.047
Platelet	0.998	0.997–1.000	0.046	1.000	0.998–1.002	0.964
pH	1.095	0.479–2.500	0.830			
BIG score	1.081	1.059–1.104	<0.001	1.111	1.084–1.14	<0.001
Surgical intervention	1.235	0.905–1.685	0.183			
Blood transfusion	1.240	0.926–1.659	0.148			
Epidural hematoma	0.592	0.172–2.036	0.405			
Subdural hematoma	1.240	0.926–1.660	0.148			
Subarachnoid hemorrhage	1.582	1.152–2.174	0.005	1.577	1.097–2.259	0.013
Intracerebral hematoma	1.663	1.130–2.450	0.010	2.121	1.358–3.289	0.001

SpO_2_, pulse oxygen saturation; OR, odds ratio; CI, confidence interval.

### Construction of the age-adjusted BIG score and multi-factor prognostic model

**Figure 2:**
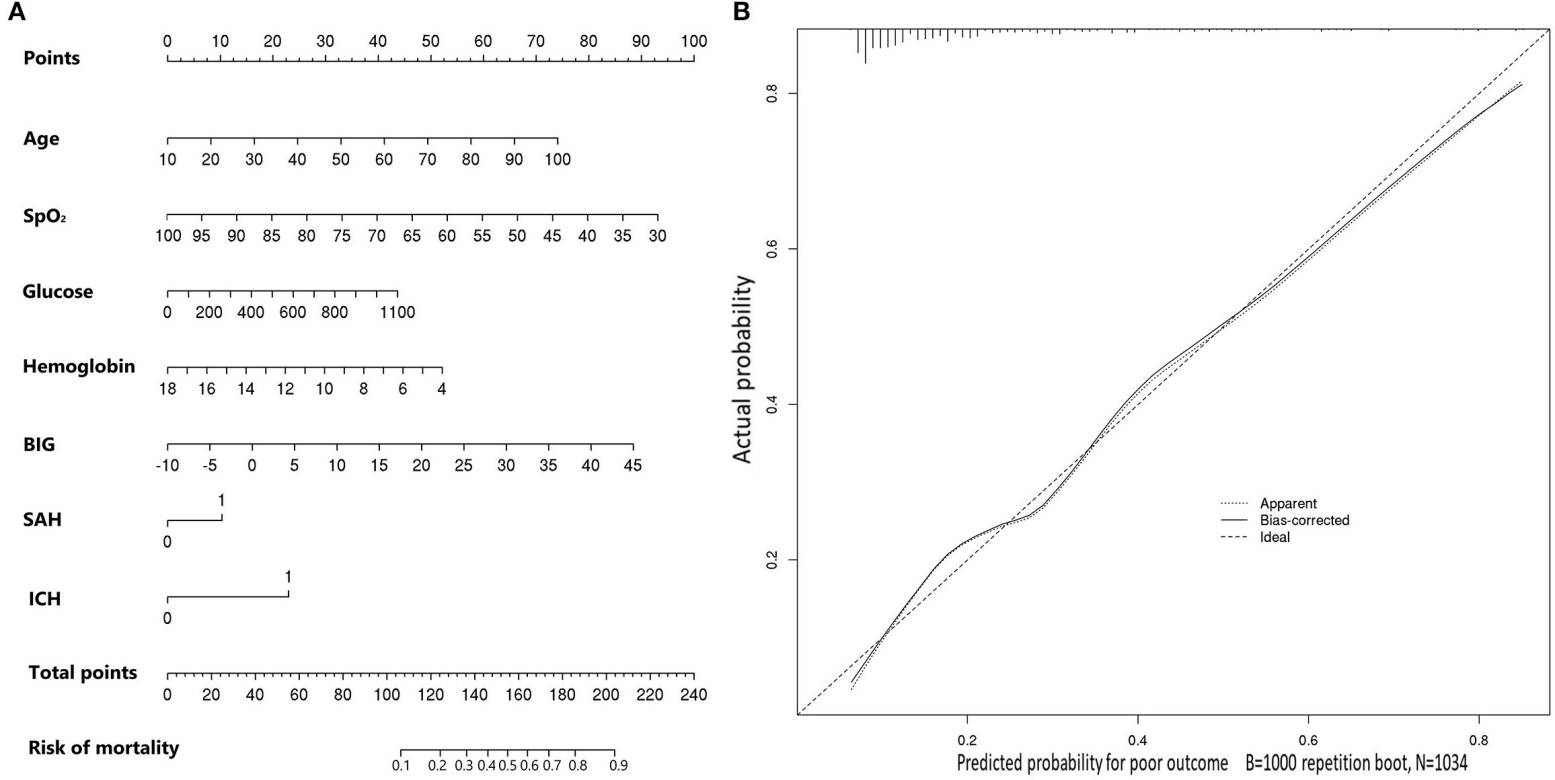
**(A)** Nomogram of the constructed prognostic model for predicting in-hospital mortality in included TBI patients. **(B)** Calibration plot of the constructed prognostic model for predicting in-hospital mortality in included TBI patients.

Multivariable logistic regression was utilized to construct an age-adjusted BIG score. Utilizing regression coefficients of age and BIG score, we calculated the age-adjusted BIG score by 0.38 × age + BIG score for convenient application. Then, a multi-factor prognostic model was also constructed by multivariable logistic regression using the abovementioned seven significant factors, which were age, SpO_2_, glucose, hemoglobin, BIG score, subarachnoid hemorrhage, and intracerebral hematoma. The nomogram of this multi-factor prognostic model was drawn to evaluate its accuracy (Figure 2A). The calibration plot showed good consistency between the actual probability and predicted probability of in-hospital mortality (Figure 2B).

### Comparison of prognostic values between BIG score, age-adjusted BIG score, and the constructed prognostic model

**Figure 3:**
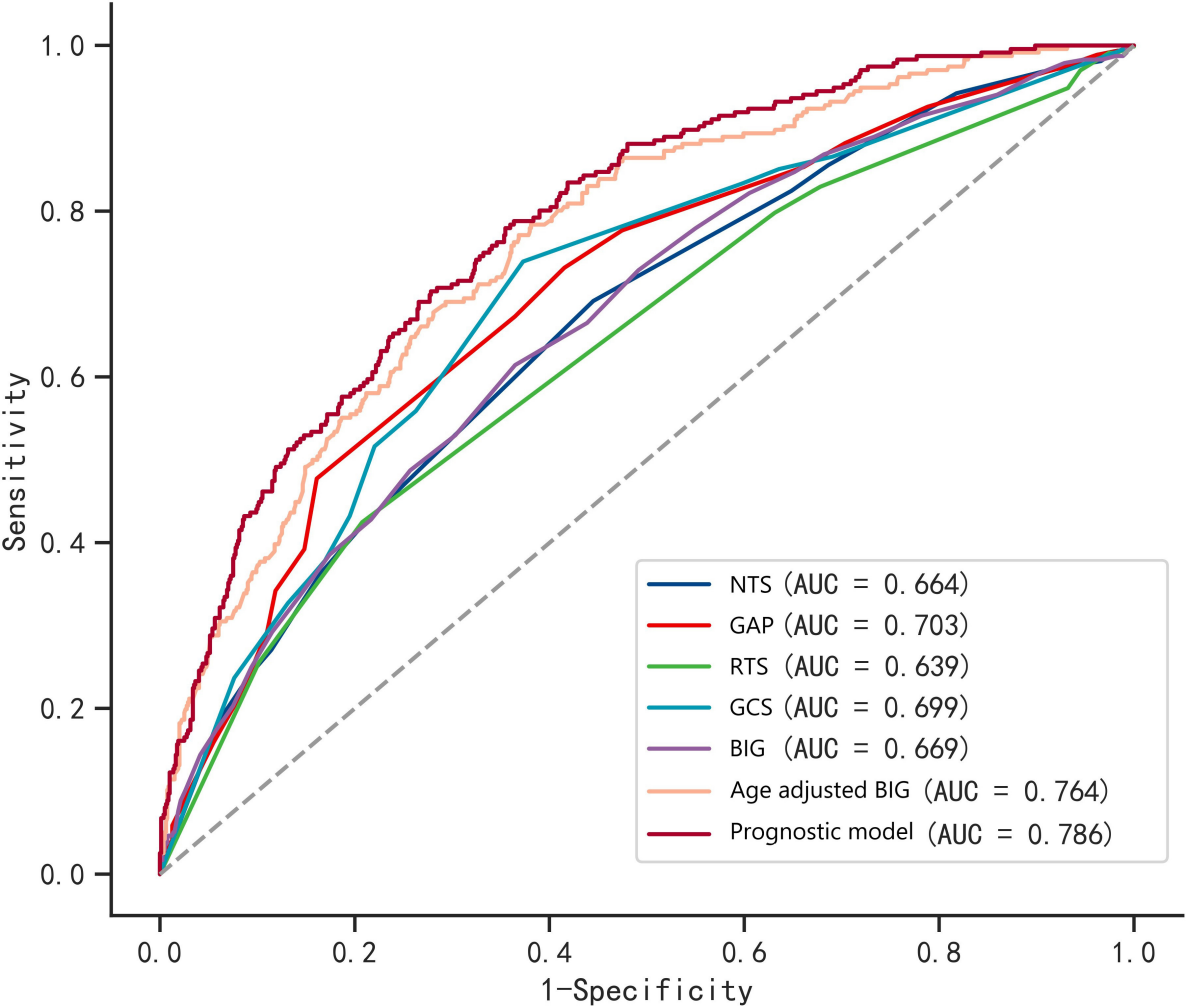
ROC curves of the BIG score, age-adjusted BIG score, other trauma triage scores, and constructed prognostic model. The constructed prognostic model is composed of age, SpO_2_, glucose, hemoglobin, BIG, subarachnoid hemorrhage, and intracerebral hematoma.

As shown in Table 3, the AUC value of single GCS and BIG scores were 0.699 and 0.669, respectively (Figure 3). The age-adjusted BIG score had higher AUC (AUC = 0.764) than GCS (*Z* = 15.795, *p* < 0.001) and BIG (*Z* = 5.352, *p* < 0.001). The constructed prognostic model composed of seven factors (age, SpO_2_, glucose, hemoglobin, BIG, subarachnoid hemorrhage, and intracerebral hematoma) had the highest AUC (AUC = 0.786).

**Table 3:** AUC value of age adjusted BIG, other trauma triage score and constructed prognostic model.

	**AUC**	**95% CI**	**Sensitivity**	**Specificity**	**Youden index**	**Best cutoff**
NTS	0.664	0.625–0.703	0.692	0.555	0.247	15
GAP	0.703	0.666–0.740	0.732	0.585	0.317	14
RTS	0.639	0.600–0.678	0.425	0.792	0.217	11
GCS	0.699	0.661–0.737	0.739	0.627	0.366	7
BIG	0.669	0.629–0.708	0.614	0.635	0.250	12
Age adjusted BIG	0.764	0.730–0.797	0.784	0.619	0.403	0.20
Prognostic model	0.786	0.754–0.818	0.703	0.722	0.425	0.24

AUC, area under the receiver operating characteristic curve; CI, confidence interval; NTS, New Trauma Score; GAP, The GCS, Age and Systolic Arterial Pressure score; RTS, Revised Trauma Score; GCS, Glasgow Coma Scale.

The prognostic model is composed of age, SpO_2_, glucose, hemoglobin, BIG, subarachnoid hemorrhage and intracerebral hematoma.

## Discussion

In our study, the BIG score was not a valuable risk stratification tool for adult TBI patients. However, the age-adjusted BIG score performed well in predicting outcomes with an AUC of 0.764. The prognostic value of the age-adjusted BIG score was superior to the readily available physiological scoring system RTS. Combining four indicators of mortality (age, base deficit, international normalized ratio, and Glasgow Coma Scale) in trauma patients, the age-adjusted BIG score could comprehensively reflect the injury severity and possible progression of adult traumatic patients.

As an indicator of shock, base deficit was confirmed to be associated with injury severity and mortality in pediatric and adult trauma patients (17–20). Researchers also concluded that base deficit was a useful predictor of coagulation decompensation and shock-related complications after trauma (21, 22). One study found that an increased base deficit was associated with prolonged partial thromboplastin and prothrombin times and low protein C levels in trauma patients (23). A Evaluating level of base deficit could help physicians decide the requirements of early transfusion to avoid the development of hypoperfusion (21, 22). Maintaining appropriate blood pressure and cerebral perfusion was necessary to alleviate secondary brain injury in TBI patients. A previous study including pediatric TBI patients illustrated that patients with poor outcomes had a higher base deficit level than those with good outcomes (12). Our results were similar to the findings of a previous study that indicated that non-survivors had a higher base deficit level than survivors. Another study showed that the base deficit on admission was statistically negatively correlated with GCS and RTS in adult TBI patients (24). However, the level of base deficit did not significantly differ between survivors and non-survivors in this study. This contradictory and unconvincing result might be attributable to the small sample size of this study. Although statistically non-significant after adjusting confounders, the base deficit was significantly related to in-hospital mortality in the univariate logistic regression analysis of our study.

It was estimated that a quarter of patients with severe trauma would present an abnormal blood coagulation test on admission, which would be positively associated with poor outcomes for these patients (25, 26). As a reflection of coagulation function, INR was confirmed as an accurate predictor of mortality and organ failure in trauma patients (27, 28). Acute traumatic coagulopathy (ATC) was primarily caused by the endothelial activation of the protein C pathway, which was induced by tissue injury and hypoperfusion (29, 30). Other factors such as resuscitation-induced hemodilution, hypothermia, and acidosis would aggravate the ATC (26, 31). The prevalence of coagulopathy after TBI had been reported as ranging from 7 to 63%. The huge discrepancy might be attributable to the different definitions of coagulopathy and heterogeneous populations (32–35). Specifically, the TBI itself was independently correlated with the development of coagulopathy due to the excessive fibrinolysis caused by extensive tissue factor release from the injured brain (36, 37). Coagulopathy was acknowledged as an independent risk factor of mortality and neurological outcomes in TBI patients (38–41). Poor coagulation function could increase the potential of intracranial hemorrhage, extracranial hemorrhage, and secondary neuronal loss (26, 36). Some studies indicated that incorporating results of the coagulation test including INR could improve the value of the conventional TBI prognostic model (42, 43). Therefore, the BIG score incorporating INR could reflect the severity and possible progression of TBI patients more comprehensively.

GCS, which has been widely used for nearly five decades, is an indicator of brain injury severity and cerebral perfusion. However, the classic GCS could not be accurately evaluated in intubated, sedated, and intoxicated patients (44, 45). The comparison of the AUC value between the GCS score alone and our age-adjusted BIG score showed that incorporating base deficit, INR, and age into GCS could improve the prognostic value and stability of clinical use in TBI patients. The multivariable prognostic model we constructed was composed of seven factors, namely, age, SpO_2_, glucose, hemoglobin, BIG score, subarachnoid hemorrhage, and intracerebral hematoma. Although this model had a significantly higher AUC value than the age-adjusted BIG score, its evaluation was much more complex than the age-adjusted BIG score, which makes it more applicable in hospitalization but not in the emergency department. Instead, the simplicity and easy availability of the age-adjusted BIG score allow it to be quickly evaluated without the consideration of additional factors. This advantage is significant for physicians carrying out patient triage and providing intensive medical therapy for potentially high-risk TBI patients in the early stage after injury. Therefore, the age-adjusted BIG score has been specially applied by emergency department workers.

There were several limitations in this study. First, most of the included patients were those who received treatment in the ICU of a single medical center. Patients with mild TBI might not be included in this study. Nearly half of TBI patients in the database who did not meet inclusion criteria were excluded. Therefore, selection bias could not be avoided. The exact predictive value of the age-adjusted BIG score and the constructed prognostic model should be externally verified by a prospective study in other medical centers. Second, the predictive value of the age-adjusted BIG score was not specifically analyzed in subgroups of included TBI patients, such as patients with a penetrating injury or blunt injury. A previous study showed that the BIG score was more valuable in predicting outcomes of penetrating trauma patients than blunt trauma patients (14). Third, the level of base deficit and INR in admission could be influenced by pre-hospital intubation and resuscitation. Records of these two variables were not collected in the present study. Finally, in addition to RTS, other complex trauma scores such as ISS and TRISS were not evaluated in the present study. A study comparing the predictive value between age-adjusted BIG scores and these scores is worthwhile to conduct in the future. Despite these limitations, the readily available age-adjusted BIG score is more efficient than other complex scores in patient triage and treatment decisions in the early stage after brain injury.

## Conclusion

As a pediatric trauma score, the BIG score is not applicable to adult TBI patients. However, the age-adjusted BIG score is a readily available and effective score that is beneficial for clinicians to triage adult TBI patients and evaluate possible progression in the early stage after injury. The prognostic model incorporating the BIG score has a better predictive value and could be used in TBI patients during hospitalization.

## Data availability statement

The original contributions presented in the study are included in the article/supplementary material, further inquiries can be directed to the corresponding authors.

## Ethics statement

The studies involving humans were approved by the Ethics Committee of the West China Hospital of Sichuan University (NO: 2021-1684). The studies were conducted in accordance with the local legislation and institutional requirements. The ethics committee/institutional review board waived the requirement of written informed consent for participation from the participants or the participants' legal guardians/next of kin because this observational study was performed using data from the Multiparameter Intelligent Monitoring in Intensive Care Database III (MIMIC-III database). All data of participants in this public database were deidentified and anonymized.

## Author contributions

XB: Conceptualization, Data curation, Methodology, Resources, Software, Validation, Writing—original draft, Writing—review & editing. RW: Conceptualization, Data curation, Methodology, Resources, Software, Validation, Writing—original draft, Writing—review & editing. CZ: Investigation, Methodology, Project administration, Writing—original draft. DW: Formal analysis, Methodology, Resources, Supervision, Validation, Writing—review & editing. LM: Data curation, Formal analysis, Methodology, Resources, Supervision, Visualization, Writing—review & editing. MH: Data curation, Formal analysis, Methodology, Supervision, Validation, Visualization, Writing—review & editing.
